# Overexpression of Tpl2 is linked to imatinib resistance and activation of MEK‐ERK and NF‐κB pathways in a model of chronic myeloid leukemia

**DOI:** 10.1002/1878-0261.12186

**Published:** 2018-04-06

**Authors:** Anna Chorzalska, Nagib Ahsan, R. Shyama Prasad Rao, Karim Roder, Xiaoqing Yu, John Morgan, Alexander Tepper, Steven Hines, Peng Zhang, Diana O. Treaba, Ting C. Zhao, Adam J. Olszewski, John L. Reagan, Olin Liang, Philip A. Gruppuso, Patrycja M. Dubielecka

**Affiliations:** ^1^ Signal Transduction Lab Division of Hematology/Oncology Rhode Island Hospital Warren Alpert Medical School Brown University Providence RI USA; ^2^ Division of Biology and Medicine COBRE CCRD Proteomics Core Facility Rhode Island Hospital Brown University Providence RI USA; ^3^ Division of Biostatistics and Bioinformatics Yenepoya Research Center Yenepoya University Mangalore India; ^4^ Cardiovascular Research Center Rhode Island Hospital Warren Alpert Medical School Brown University Providence RI USA; ^5^ Department of Biostatistics Yale School of Public Health New Haven CT USA; ^6^ Flow Cytometry and Cell Sorting Core Facility Roger Williams Medical Center Providence RI USA; ^7^ Department of Pathology and Laboratory Medicine Rhode Island Hospital Warren Alpert Medical School Brown University Providence RI USA; ^8^ Cardiovascular Lab Department of Surgery Roger Williams Medical Center Boston University School of Medicine Providence RI USA; ^9^ Division of Hematology/Oncology Rhode Island Hospital Warren Alpert Medical School Brown University Providence RI USA; ^10^ Department of Pediatrics Rhode Island Hospital Brown University Providence RI USA

**Keywords:** Bcr‐Abl1, COT1, imatinib resistance, Map3k8, stem cells, Tpl2

## Abstract

The introduction of tyrosine kinase inhibitors (TKI) has transformed chronic myeloid leukemia (CML) into a chronic disease with long‐term survival exceeding 85%. However, resistance of CML stem cells to TKI may contribute to the 50% relapse rate observed after TKI discontinuation in molecular remission. We previously described a model of resistance to imatinib mesylate (IM), in which K562 cells cultured in high concentrations of imatinib mesylate showed reduced Bcr‐Abl1 protein and activity levels while maintaining proliferative potential. Using quantitative phosphoproteomic analysis of these IM‐resistant cells, we have now identified significant upregulation of tumor progression locus (Tpl2), also known as cancer Osaka thyroid (COT1) kinase or Map3k8. Overexpression of Tpl2 in IM‐resistant cells was accompanied by elevated activities of Src family kinases (SFKs) and NF‐κB, MEK‐ERK signaling. CD34+ cells isolated from the bone marrow of patients with CML and exposed to IM *in vitro* showed increased *MAP3K8* transcript levels. Dasatinib (SFK inhibitor), U0126 (MEK inhibitor), and PS‐1145 (IκB kinase (IKK) inhibitor) used in combination resulted in elimination of 65% of IM‐resistant cells and reduction in the colony‐forming capacity of CML CD34+ cells in methylcellulose assays by 80%. In addition, CML CD34+ cells cultured with the combination of inhibitors showed reduced *MAP3K8* transcript levels. Overall, our data indicate that elevated Tpl2 protein and transcript levels are associated with resistance to IM and that combined inhibition of SFK, MEK, and NF‐κB signaling attenuates the survival of IM‐resistant CML cells and CML CD34+ cells. Therefore, combination of SFK, MEK, and NF‐κB inhibitors may offer a new therapeutic approach to overcome TKI resistance in CML patients.

**Abbreviations**
ABL1: Abelson kinase 1
BCR: break point cluster region protein
CML: chronic myeloid leukemia
COT1: cancer Osaka thyroid 1 kinase
ERK: extracellular signal‐regulated kinase
IKK: IκB kinase
IM: imatinib mesylate
MEK: mitogen‐activated protein/extracellular signal‐regulated kinase kinase
NF‐κB: nuclear factor‐κB
SFK: Src family kinases
TKI: tyrosine kinase inhibitors
TPL2: tumor progression locus 2

## 1 Introduction

Chronic myeloid leukemia (CML) is a clonal myeloid neoplasm originating from malignant hematopoietic stem cells (HSCs) that express constitutively active, oncogenic Bcr‐Abl1 kinase. *BCR‐ABL1* is a product of a reciprocal translocation between chromosomes 9 and 22 t(9:22) resulting in a fusion of the break point cluster region protein (*BCR*) and Abelson kinase (*ABL1*) genes (Jabbour and Kantarjian, 2016). With the discovery of a specific and potent tyrosine kinase inhibitor (TKI), imatinib, and its derivatives, dasatinib, nilotinib, bosutinib, and ponatinib (Druker *et al*., 1996; Golas *et al*., 2003; O'Hare *et al*., 2009; Shah *et al*., 2004; Weisberg *et al*., 2005), CML became a manageable chronic disease with a long‐term survival exceeding 85%. However, CML patients remain on the lifelong TKI treatment associated with adverse effects that diminish quality of life (Experts in Chronic Myeloid, 2013). In addition, up to 25% of CML patients in the chronic phase experience disease progression during therapy with TKIs. While about 50% of these patients have mutations in the Bcr‐Abl1 kinase domain (Castagnetti *et al*., 2017), it remains unclear why patients without these mutations lose their response to therapy.

Three major clinical trials evaluating the effect of discontinuing TKI therapy in CML patients who achieve a durable, complete molecular remission reported a relapse rate of about 50% within 6–7 months of treatment discontinuation (Imagawa *et al*., 2015; Mahon *et al*., 2010; Ross *et al*., 2013). The current consensus is that rare Bcr‐Abl1‐expressing HSCs (referred here to as CML stem cells) are insensitive to TKIs and are the cause of the disease relapse after TKI discontinuation. Extensive efforts have been made to identify unique molecular and signaling signatures of CML stem cells, allowing their targeted eradication. It has been established that CML stem cells remain independent from the Bcr‐Abl1 kinase activity for their survival and therefore are not ‘oncogene addicted’ (Corbin *et al*., 2011; Hamilton *et al*., 2012; Koschmieder *et al*., 2005). Several key signaling pathways have been identified as crucial for CML stem cell self‐renewal and survival in the presence of TKIs (reviewed in Holyoake and Vetrie (2017)). These include PI3K/AKT/FOXO (Naka *et al*., 2010; Pellicano *et al*., 2014), Wnt/β‐catenin (Heidel *et al*., 2012; Hu *et al*., 2009; Zhao *et al*., 2007), JAK/STAT (Eiring *et al*., 2015; Gallipoli *et al*., 2014; Traer *et al*., 2012), and Hedgehog/SMO (Irvine *et al*., 2016; Zhao *et al*., 2009). The role of Bcr‐Abl1 signaling in the regulation of these pathways in CML stem cells remains unclear, and the effect of long‐term TKI exposure on these cascades is not understood. Notably, recent reports indicate that prolonged treatment with TKI supports stem cell‐like characteristics of primitive, leukemia‐initiating CML stem cells (Charaf *et al*., 2017; Chorzalska *et al*., 2017).

Src family kinases (SFKs) have been implicated in both Bcr‐Abl1‐dependent and Bcr‐Abl1‐independent mechanisms of resistance to TKIs (Danhauser‐Riedl *et al*., 1996; Donato *et al*., 2003; Grosso *et al*., 2009; Hu *et al*., 2004; Irwin *et al*., 2015; Pene‐Dumitrescu and Smithgall, 2010; Warmuth *et al*., 1997). SFK transcript levels were found to be elevated in more than 50% of CML patients unresponsive to TKIs (Hayette *et al*., 2011), and inhibition of SFKs was shown to significantly improve eradication of *BCR‐ABL1*‐positive leukemia cells in mice (Hu *et al*., 2006). However, inhibition of Src kinase activity and its downstream signaling pathways in human CML progenitors had only limited pro‐apoptotic effect (Konig *et al*., 2008a). While available evidence supports the importance of SFK signaling in systemic maintenance of CML stem cells, SFK inhibition alone seems to be insufficient to eradicate these cells.

The Ras‐Raf‐MEK‐ERK pathway is one of the central, microenvironmentally tuned signaling cascades regulating cell proliferation, differentiation, survival, and motility. This pathway has also been implicated in drug sensitivity and chemoresistance. In this pathway, growth factor/cytokine/mitogen stimulation of a cognate receptor leads to recruitment of the adapter protein Shc, which in turn recruits growth factor receptor bound protein 2 (Grb2) and son of sevenless homolog protein (Sos), resulting in activation and GTP binding of membrane‐bound H‐, K‐, or N‐Ras GTPase. Activated Ras recruits A‐, B‐, or C‐Raf homo‐ or heterodimers to the membrane, where they become activated *inter alia* by SFKs. Activated Raf then phosphorylates and activates MEK, which in turn phosphorylates and activates ERK1/2. These terminal kinases have more than 60 targets that exert potent effects on cell growth and survival (von Kriegsheim *et al*., 2009; Samatar and Poulikakos, 2014). Some reports indicate that Ras‐Raf‐MEK‐ERK pathway can be activated by classical chemotherapy drugs used in leukemia therapy (McCubrey *et al*., 2006, 2008; Steelman *et al*., 2011a,b). Furthermore, it was shown that nilotinib can induce an increase in ERK activity in CML CD34+ cells (Konig *et al*., 2008b); however, the detailed mechanism by which MEK‐ERK activation occurs in CML stem cells upon exposure to TKI remains to be elucidated.

NF‐κB signaling is also significantly upregulated in leukemic stem cells subjected to chemotherapy (Zhou *et al*., 2015). Mammals express five NF‐κB proteins: RelA (p65), RelB, c‐Rel, NF‐κB1, and NF‐κB2. NF‐κB1 and NF‐κB2 are synthesized as large precursors of 105 (p105) and 100 kDa (p100), which are cleaved to produce transcriptionally active p50 and p52 subunits, respectively. The canonical NF‐κB pathway depends on activation of the IKK (inhibitor κB[IκB] kinase) complex, which consists of IKKα, IKKβ, and NF‐κB essential modulator (NEMO or IKKγ). Activation of this complex leads to phosphorylation and rapid degradation of IκB, which in turn releases and activates NF‐κB. The noncanonical NF‐κB pathway involves activation of NIK (NF‐κB‐inducing kinase) and IKK to induce processing of p100 to transcriptionally active p52. In addition, phosphorylation of p105 by the classical IKK complex triggers its polyubiquitination, degradation, and release of transcriptionally active p50.

A protein kinase that links MEK‐ERK and NF‐κB signaling is tumor progression locus (Tpl2), also known as cancer Osaka thyroid (COT1) or Map3k8. In unstimulated cells, Tpl2 remains associated with NF‐κB1. This interaction inhibits both Tpl2 kinase activity and protects NF‐κB1 from proteolytic processing and activation (Beinke and Ley, 2004; Cho and Tsichlis, 2005; Gantke *et al*., 2011; Yang *et al*., 2012). Phosphorylation of p105 on Ser927 and 932 by IKKβ drives the dissolution of this complex, targeting NF‐κB1 for ubiquitination and proteasomal degradation and release of transcriptionally active p50 (Beinke and Ley, 2004; Cho and Tsichlis, 2005; Gantke *et al*., 2011; Yang *et al*., 2012). Dissociation and degradation of NF‐κB1 free Tpl2, leading to activation of downstream pathways that include MEK‐ERK and its downstream effectors (Beinke and Ley, 2004; Cho and Tsichlis, 2005; Gantke *et al*., 2011; Yang *et al*., 2012). Overexpression of Tpl2 has been shown to drive resistance to Raf inhibition in melanoma (Johannessen *et al*., 2010). The role of Tpl2 in chemoresistance of leukemic stem cells remains to be explored.

Here, we present evidence that Tpl2 is overexpressed in our CML cellular model of resistance to imatinib mesylate (IM) and that *MAP3K8*, together with *NFKB1* transcript levels, are significantly elevated in CML CD34+ cells exposed to IM. Overexpression of Tpl2 is accompanied by increased activity of SFKs, MEK‐ERK, and NF‐κB in IM‐resistant cells. We show for the first time that combination of SFK, MEK, and NF‐κB cascade inhibitors significantly reduces survival of IM‐resistant cells and IM‐insensitive CML CD34+ cells. Combined inhibition of SFK, MEK, and NF‐κB pathways may present a new therapeutic option to target CML stem cells unresponsive to IM therapy.

## 2 Materials and methods

### 2.1 CD34+ cells isolation and culture

Bone marrow cells were obtained from patients (*n* = 3) with p210 *BCR‐ABL1*‐positive CML before initiation of IM therapy. Informed consent was obtained in accordance with the Declaration of Helsinki, and the procedures were approved by the Institutional Research Board at Rhode Island Hospital. CD34+ cell‐enriched populations (> 85% purity) were isolated before cryopreservation using a MicroBeads‐based kit from Miltenyi Biotec (Cambridge, MA, USA). For suspension cultures, CD34+ cells were suspended in StemSpan serum‐free expansion medium (SFEM II; Stemcell Technologies, Vancouver, BC, Canada) supplemented with 10 ng·mL^−1^ GM‐CSF, 10 ng·mL^−1^ G‐CSF, 100 ng·mL^−1^ SCF, 50 ng·mL^−1^ LIF, 100 ng·mL^−1^ MIP‐1α, and 10 ng·mL^−1^ IL‐6. A total of 5 × 10^4^ cells were then added to each well of a 24‐well plate with or without 5 μm IM. The number of apoptotic cells was evaluated by FACS (LSR II, facsdiva software v8.0; Becton Dickinson, San Jose, CA, USA) after 1 week of incubation using the Annexin V‐FITC Early Apoptosis Detection Kit (#6592) from Cell Signaling Technology (Danvers, MA, USA). For *in vitro* colony assays, 2 × 10^3^ CD34+ cells were plated in quadruplicate in methylcellulose‐based medium with recombinant cytokines SCF, IL‐3, EPO, GM‐CSF (#H4434; Stem Cell Technologies) in the presence of 5 μm U0126, 50 nm dasatinib, 10 μm PS‐1145, 50 nm dasatinib + 5 μm U0126, 50 nm dasatinib + 10 μm PS‐1145, 50 nm dasatinib + 5 μm U0126 + 10 μm PS‐1145 (Cayman Chemicals, Ann Arbor, MI, USA). Colonies derived from burst‐forming units erythroid (BFU‐E), multilineage granulopoietic, erythroid, macrophage, and megakaryocytic colony‐forming units (CFU‐GEMM), granulocyte–macrophage colony‐forming units (CFU‐GM), and macrophage colony‐forming units (CFU‐M) were scored *in situ* after 14 days of incubation using an inverted microscope.

### 2.2 Cell lines and cell culture

The human CML K562 cell line and its IM‐resistant counterpart, clone K562‐STI‐R, were established and grown as described previously (Chorzalska *et al*., 2017). K562 and K562‐STI‐R cell lines are routinely tested for the presence of the hematopoietic cell marker CD45 by FACS and for the presence of Bcr‐Abl1 oncogene by immunoblotting. Authenticity of both K562 and K562‐STI‐R was also determined by the short tandem repeat profiling. Cells in a logarithmic growth phase were used for all experiments. For drug sensitivity experiments, cells were grown in 0.6 μm IM, 25 μm IM, 25 μm U0126, 100 nm dasatinib, 25 μm PS‐1145, combinations of 25 μm IM + 25 μm U0126, 100 nm dasatinib + 25 μm U0126, 100 nm dasatinib + 25 μm PS‐1145, and 100 nm dasatinib + 25 μm U0126 + 25 μm PS‐1145. p58‐overexpressing K562 cells (sorted based on GFP expression 24 h postelectroporation) were cultured in the presence of 1, 10, or 25 μm of IM. The effects of all treatments were evaluated after an incubation period of 16 h. The number of apoptotic cells was determined by FACS using the Annexin V‐FITC Early Apoptosis Detection Kit.

### 2.3 Plasmid constructs, electroporation, and cell sorting

gBlock gene fragment encoding the open‐reading frame of human p58 (ENSG00000107968) with a 5′ BamHI site followed by a Kozak sequence (GCCACC) and a 3′ XhoI site was ordered from Integrated DNA Technologies (Coralville, IA, USA). BamHI/XhoI‐digested fragment was cloned into BamHI/XhoI‐cut pENTR‐CMV‐HBG‐3xHA‐IRES‐hrGFP vector kindly provided by Gideon Koren (Rhode Island Hospital, Cardiovascular Research Center, Providence, RI). The resulting sequence‐verified vector pENTR‐CMV‐HBG‐p58‐3xHA‐IRES‐hrGFP allows coexpression of HA‐tagged p58 and humanized *Renilla reniformis* GFP. Detailed map of the used vector is presented in Fig. S1A. Control and p58‐expressing vectors used for electroporation were purified using EndoFree Plasmid Maxi Kit (Qiagen GmbH, Hilden, Germany). DNA electroporation was performed using Neon^®^ Transfection System (Life Technology, Carlsbad, CA, USA) according to optimized manufacturer's instruction. After electroporation, cells were plated and GFP‐positive cells were sorted after 24 h. Cell sorting was performed using a BD Influx cell sorter (BD Biosciences, San Jose, CA, USA). Electroporation efficiency was determined as 70% for the control GFP‐expressing cells and 56–59% for p58 and GFP‐expressing cells. Sorting data for three independent K562 electroporation experiments are presented on Fig. S1B.

### 2.4 Quantitative RT/PCR analysis

Total RNA from CD34+ cells cultured in the presence of 5 μm IM was purified using an RNeasy Plus Mini Kit (Qiagen Hilden, Germany). RT/PCR was performed as described (Chorzalska *et al*., 2017). The following primers were designed for transcript quantification: *MAP3K8*: For: 5′ATGGAGTACATGAGCACTGGA3′ and Rev: 5′GCTGGCTCTTCACTTGCATAAAG3′; *NFKB1*: For: 5′AACAGAGAGGATTTCGTTTCCG3′ and Rev: 5′TTTGACCTGAGGGTAAGACTTCT3′; *ACTB*: For: 5′CATGTACGTTGCTATCCAGGC3′ and Rev: 5′CTCCTTAATGTCACGCACGAT3′, S18 rRNA: For: 5′GGGCGGAGATATGCTCATGTG3′ and Rev: 5′TCTGGGATCTTGTACTGTCGT3′. All primers were purchased from IDT DNA. Results were normalized to the level of human *ACTB* and S18 rRNA for CML CD34+ cells and S18 rRNA for K562 cell lines. Relative quantitation of gene expression was evaluated by CFX96™ Real‐Time System (Bio‐Rad, Hercules, CA, USA). Data were analyzed using the Bio‐Rad CFX Manager (Bio‐Rad).

### 2.5 Phosphopeptide enrichment, MS/MS analysis and relative quantitation of the phosphopeptides

K562 or K562‐STI‐R cells in a logarithmic growth phase were subjected to phosphopeptide enrichment and proteomic analysis as described previously (Chorzalska *et al*., 2017). Five independently revived and expanded cellular clones per cell line were harvested, lysed, and used to prepare tryptic digested peptides. The peptides were lyophilized, and phosphopeptide enrichment was performed using Titansphere Phos‐TiO tips (GL Sciences, Tokyo, Japan) following the manufacturer's protocol with minor modifications (Ahsan *et al*., 2017). LC‐MS/MS was performed on a fully automated proteomic technology platform (Yu and Salomon, 2010) that utilizes an Agilent 1200 Series Quaternary HPLC system (Agilent Technologies, Santa Clara, CA, USA) connected to a Q Exactive Plus mass spectrometer (Thermo Fisher Scientific, Waltham, MA, USA). MS/MS spectra were searched against a human‐specific database (UniProt; downloaded 2/1/2013) using mascot v. 2.4 (Matrix Science, Ltd, London, UK). Peptide assignments from the database search were filtered down to 1% false discovery rate (FDR) by a logistic spectral score, as previously described (Chorzalska *et al*., 2017; Elias and Gygi, 2007; Yu *et al*., 2009). To validate the position of phosphorylation sites, the Ascore algorithm (Beausoleil *et al*., 2006) was applied and the reported phosphorylation site position reflected the top Ascore prediction. Relative quantification of phosphopeptides abundance was performed via calculation of selected ion chromatograms (SIC) peak areas. Retention time alignment of individual replicate analyses was performed as previously described (Demirkan *et al*., 2011).

### 2.6 Immunoblotting

Whole‐cell lysates were used for all immunoblotting analyses. Protein levels were normalized to histone H3 content. Western blotting was performed as described previously (Chorzalska *et al*., 2014). The signal generated by peroxidase‐conjugated antibodies was detected using the Femto chemiluminescent substrate (for ERK1, pIkBα, Tpl2, B‐Raf) or SuperSignal West Pico Plus (for all other antigens; ThermoFisher Scientific, Franklin, MA, USA). The following antibodies were used: rabbit polyclonal anti‐Tpl2 #ab137589 (Abcam Inc., Cambridge, MA, USA), mouse monoclonal anti‐Tpl‐2 #sc‐373677, mouse monoclonal anti‐Abin‐2 #sc‐271850, rabbit polyclonal anti‐Hck #sc‐72 (Santa Cruz Biotechnology, Dallas, TX, USA), rabbit monoclonal anti‐NF‐κB1 p105/p50 #13586, rabbit monoclonal anti‐phospho‐NF‐κB1 p105 (Ser 933) #4806, rabbit monoclonal anti‐NF‐κB p65 #8242, rabbit monoclonal anti‐phospho‐NF‐κB p65 (Ser 536) #3033, mouse monoclonal anti‐IKKα #11930, rabbit monoclonal anti‐IKKβ #8943, rabbit monoclonal anti‐phospho‐IKKα/β (Ser 176/180) #2697, rabbit monoclonal anti‐histone H3 #4499, rabbit polyclonal anti‐A‐Raf #4432, rabbit polyclonal anti‐phospho‐A‐Raf (Ser 299) #4431, rabbit monoclonal anti‐B‐Raf #9433, rabbit polyclonal anti‐phospho‐B‐Raf (Ser 445) #2696, mouse monoclonal anti‐c‐Raf #12552, rabbit polyclonal anti‐phospho‐c‐Raf (Ser 259) #9421, rabbit polyclonal anti‐phospho‐c‐Raf (Ser 289/296/301) #9431, rabbit monoclonal anti‐phospho‐c‐Raf (Ser 338) #9427, mouse monoclonal anti‐MEK1 #2352, rabbit polyclonal anti‐MEK2 #9125, rabbit monoclonal anti‐phospho‐MEK1/2 (Ser 217/221) #9154, rabbit polyclonal anti‐ERK1 #4372, rabbit polyclonal anti‐ERK2 #9108, rabbit polyclonal anti‐ERK1/2 #9102, rabbit polyclonal anti‐phospho‐ERK1/2 (Thr 202/Tyr 204) #9101, rabbit monoclonal anti‐Src #2123, rabbit polyclonal anti‐phospho‐Src (Tyr 416) #6943, rabbit polyclonal anti‐phospho‐Src (Tyr 527) #2105, rabbit polyclonal anti‐Lck #2752, rabbit polyclonal anti‐Fyn #4023, rabbit polyclonal anti‐Fgr #2755, rabbit monoclonal anti‐Lyn #2796, horseradish peroxidase‐conjugated AffiniPure horse anti‐mouse (H+L) and goat anti‐rabbit (H+L) for chemiluminescent detection were all from Cell Signaling Technology.

### 2.7 Statistical analyses

**Figure 1:**
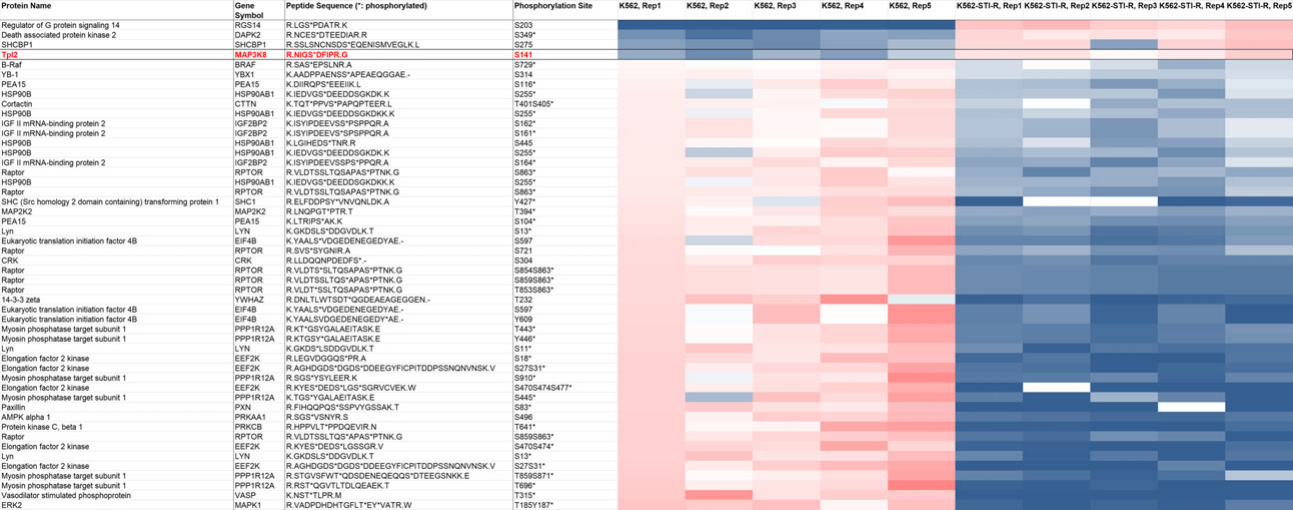
Heatmap representing abundance of phosphopeptides derived from MAPK‐ERK pathway‐specific core interactors. Abundance of phosphopeptides derived from 275 proteins listed in the MAPK‐Erk Pathway SuperPath was determined within the list of 2593 phosphopeptides displaying significant differences in IM‐sensitive K562 vs. IM‐resistant K562‐STI‐R cells. To select phosphopeptides that show statistically significant change in abundance between K562‐STI‐R and K562 cell lines, two‐tailed unpaired Student's *t*‐test and *q*‐values for multiple hypothesis tests were calculated based on the determined *P*‐values using the R package QVALUE. A heat map was generated by calculating a mean peak area for all samples showing statistical differences and then determining the ratio of individual peak areas to the means. Ratios range from 0.2 (blue) to 5 (red), 1 = white.

To evaluate the significance of differences across multiple conditions, one‐way analysis of variance was performed using SigmaPlot (Systat, San Jose, CA, USA). The Holm–Sidak post hoc test was used for multiple comparisons vs. a designated control group. Differences in mean values among the treatment groups with *P*‐values < 0.05 were considered significant. Throughout the article, the symbol * indicates *P* < 0.05, ** indicates *P* < 0.01, and *** indicates *P* < 0.001. For phosphoproteomic analyses, *P*‐values were calculated from five replicates. To select phosphopeptides that showed a statistically significant change in abundance between K562‐STI‐R and K562 cell lines (Fig. 1, Tables S1 and S2) two‐tailed unpaired Student's *t*‐test and *q*‐values for multiple hypothesis tests were calculated based on the determined *P*‐values using the R package QVALUE as previously described (Storey and Tibshirani, 2003).

## 3 Results

### 3.1 Phosphoproteomic analysis of dysregulated signaling in IM‐resistant cells

We previously described a Bcr‐Abl1‐independent model of resistance to IM (K562‐STI‐R) in which CML K562 cells cultured in high concentration of IM displayed reduced Bcr‐Abl1 protein and activity levels, and could withstand high concentrations of IM (Chorzalska *et al*., 2014). In addition, exposure of K562‐STI‐R cells to IM resulted in significantly decreased phosphorylation of Bcr‐Abl1 substrates and effectors Crk, CrkL, and STAT5 (Chorzalska *et al*., 2014). Quantitative phosphoproteomic analysis of these IM‐resistant cells identified 2593 phosphopeptides displaying significant differences in abundance compared with IM‐sensitive K562 cells (Table S1). Analysis using metacore™ software (Thompson Reuters, New York, NY, USA) identified MEK‐ERK signaling as the third most dysregulated pathway after cell cycle (chromosome condensation) and cytoskeleton remodeling pathways (Chorzalska *et al*., 2017), with *P*‐value of 1.225e‐08 and FDR of 2.774e‐06. Within the list of phosphopeptides showing statistically significant difference in abundancies between K562 and K562‐STI‐R cells, we assessed the abundance of 275 MEK‐ERK pathway‐specific core interactor peptides (275 genes listed in the MAPK‐ERK Pathway SuperPath http://pathcards.genecards.org/Pathway/273) and identified the Tpl2‐derived phosphopeptide as the fourth most abundant in IM‐resistant cells (Fig. 1, Table S2). Also, apparent in the phosphoproteomic data was reduced abundance of Crk and STAT5‐derived phosphopeptides, loss of A‐, B‐, and C‐Raf‐derived phosphopeptides, and an increased abundance of Tpl2‐derived phosphopeptides.

### 3.2 Expression of *MAP3K8* and *NFKB1* are increased IM‐resistant cells

**Figure 2:**
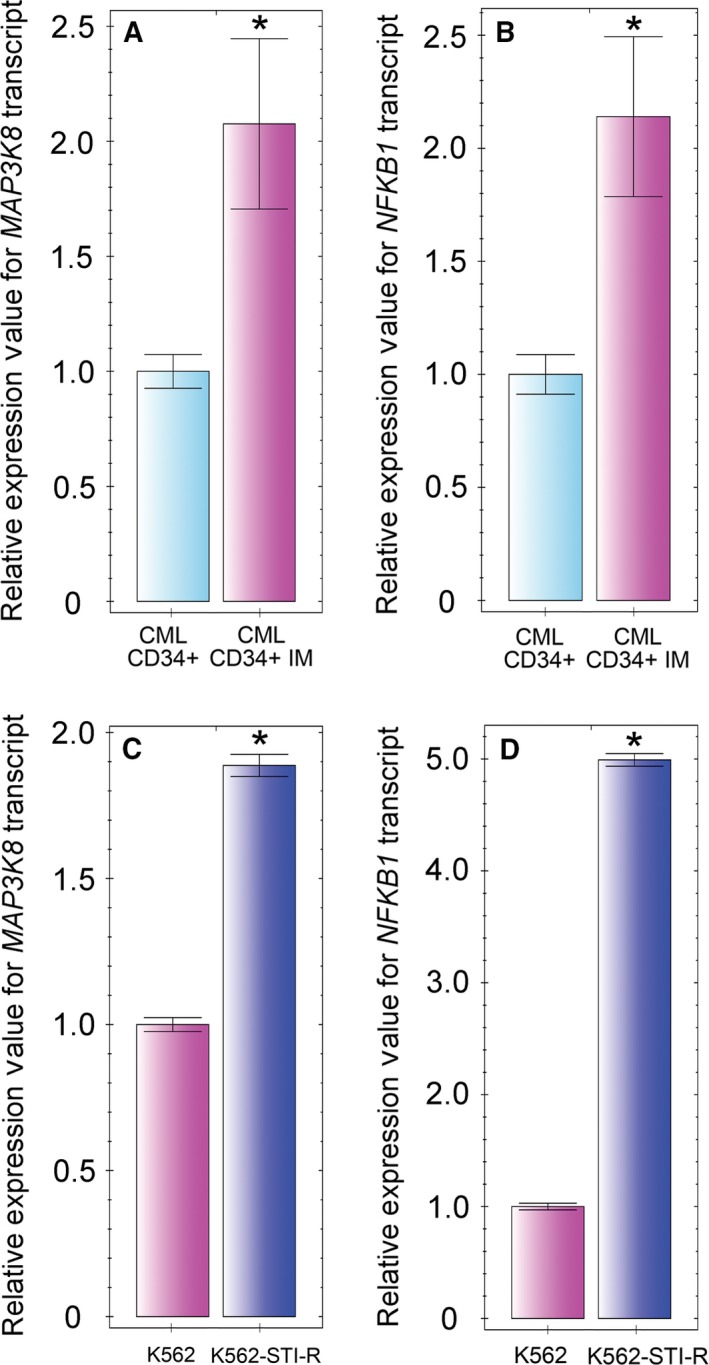
Imatinib mesylate effect on *MAP3K8 and NFKB1* expression in hematopoietic stem/progenitor cells obtained from CML patients and CML cell lines. Hematopoietic stem/progenitor cells were obtained from Bcr‐Abl1+ CML patients (*n* = 3) prior to initiation of TKI therapy. *MAP3K8* (A) and *NFKB1* (B) transcript levels were determined in the CML CD34+ cells cultured with or without 5 μm IM for 7 days. *MAP3K8* (C) and *NFKB1* (D) transcript levels were determined in K562 and K562‐STI‐R cells from three independent cell culture experiments. Data are shown as normalized expression relative to 18S rRNA and ACTB (A, B) or to 18S rRNA (C, D), expressed as fold‐change. Samples were cultured and analyzed in triplicate. *P < 0.05.

Based on the published observation that overexpression of Tpl2 drives chemoresistance in melanoma (Johannessen *et al*., 2010), and the observed abundance of Tpl2‐derived phosphopeptides in our Bcr‐Abl1‐independent model of IM resistance, we evaluated transcript levels of *MAP3K8* in CML CD34+ cells subjected to a 7‐day exposure to 5 μm IM (Corbin *et al*., 2011; Holtz *et al*., 2005; Jiang *et al*., 2010). These cells showed a twofold increase in *MAP3K8* transcripts and a 1.7‐fold increase in the transcript levels of the Tpl2‐binding partner, *NFKB1* (Fig. 2A, B). Notably, we also observed twofold and fivefold elevations of the levels of *MAP3K8* and *NFKB1* transcripts, respectively, in IM‐resistant K562‐STI‐R cells relative to K562 control cells (Fig. 2C, D).

### 3.3 NF‐κB, MEK‐ERK, and SFK signaling is upregulated in Tpl2‐overexpressing cells exhibiting Bcr‐Abl1‐independent resistance to IM

**Figure 3:**
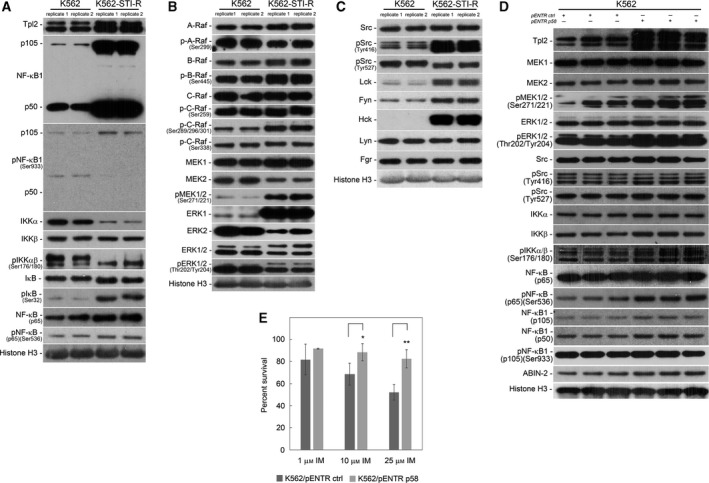
Status of NF‐κB, MEK‐ERK, and SFK signaling in IM‐resistant cells. Western blot analyses were performed on K562 and K562‐STI‐R whole‐cell lysates. Protein levels and phosphorylation status of (A) NF‐κB pathway components (Tpl2, NF‐κB1 (p105, p50), IKKα and β, IkB, and NF‐κB (p65)), (B) Raf‐MEK‐ERK pathway components (A‐, B‐, C‐Raf, MEK1 and 2, ERK 1 and 2) and (C) SFKs were evaluated. Representative immunoblots of three independent experiments are shown. Histone H3 was used as a loading control. The densitometry results of the western blots along with statistical analyses are summarized in Fig. S2 and S6A. (D) Protein levels and phosphorylation status of MEK‐ERK pathway components (MEK1 and 2, ERK 1 and 2), SFKs and NF‐κB pathway components (Tpl2, NF‐κB1 (p105, p50), ABIN‐2, IKKα and β and NF‐κB (p65)) were evaluated in Tpl2 p58‐overexpressing K562 cells. K562 cells were electroporated with the control and Tpl2 p58‐expressing vectors, GFP‐positive cells were sorted after 24 h, and whole‐cell lysates were collected and analyzed. Data from three independent electroporation experiments are shown. Histone H3 was used as a loading control. (E) FACS analysis of the percentage of Annexin V‐FITC‐positive (apoptotic) cells in a population of Tpl2 p58‐overexpressing K562 cells cultured in the presence of 1, 10, and 25 μm of IM. Data are shown as the mean and 1 SD for three independent analyses. *P < 0.05, **P < 0.01.

To further explore the involvement of Tpl2 and NF‐κB1 in Bcr‐Abl1‐independent resistance to IM, we determined the status of their expression and the activity of NF‐κB signaling in our cellular model of IM resistance. Protein levels of Tpl2 (p52 and p58 isoforms) and of NF‐κB1 were increased in K562‐STI‐R cells (Fig. 3A). Unprocessed p105 NF‐κB1 and the proteolytically cleaved, transcriptionally active p50 forms of NF‐κB1 were both elevated in K562‐STI‐R cells. The increase in phosphorylation of the IKKβ‐specific site on NF‐κB1 p105, Ser933, in IM‐resistant cells appeared to be due to an increase in the content of this protein rather than higher stoichiometry of phosphorylation.

Given that the presence of the p50 form of NF‐κB1 is likely associated with activation of the NF‐κB cascade, we evaluated the activity of major components of this pathway (Fig. 3A). In IM‐resistant cells, we observed decreased protein levels of IKKα (85 kDa), unchanged protein levels of IKKβ (87 kDa), and an increase in the phosphorylation of Ser177/Ser181 on IKKα/β at the molecular mass corresponding to IKKα, consistent with IKKα activation. We also observed an increase in the phosphorylation of IkB on Ser32, an IKK‐mediated phosphorylation site that triggers the proteasomal processing and activation of NF‐κB p65. Finally, the IM‐resistant cells also showed an increase in IKK‐mediated phosphorylation of NF‐κB p65 on Ser536, consistent with its activation. These data indicate activation of NF‐κB signaling in Tpl2‐overexpressing IM‐resistant K562‐STI‐R cells.

Considering the apparent activation of MEK‐ERK signaling in the IM‐resistant cells, the difference in Tpl2 expression, and the known effect of its overexpression on activation of MEK‐ERK signaling (Gantke *et al*., 2011; Johannessen *et al*., 2010), we assessed the status of this signaling module in K562‐STI‐R cells (Fig. 3B). The protein levels of A‐, B‐, and C‐Raf, as well as their phosphorylation status on Ser299, Ser445, and Ser338, respectively, were unchanged in the IM‐resistant cells relative to the K562 cell line. However, phosphorylation of C‐Raf at Ser289, Ser296, and Ser301, inhibitory sites associated with MEK‐ERK negative feedback inhibition of C‐Raf activity (Dougherty *et al*., 2005), was greater in the IM‐resistant cells (Fig. 3B, Fig. S2A). Evaluation of the activity status of Raf downstream effectors showed no difference in MEK1 abundance. However, MEK2 abundance was decreased in the K562‐STI‐R cells, and MEK1/2 phosphorylation at Ser271/221 was markedly enhanced. We also observed markedly higher ERK1 content and lower ERK2 content. There was no clear change in the relative stoichiometry of ERK1/ERK2 phosphorylation at Thr202/Tyr204 (Fig. 3B, Fig. S2B). Nonetheless, these data are consistent with Raf‐independent activation of MEK in Tpl2‐overexpressing K562‐STI‐R cells.

To identify upstream mediators of MEK phosphorylation other than Raf, we evaluated the activity of SFKs, which are often overexpressed/overactive in Bcr‐Abl1‐positive leukemia cells (Fig. 3C, Fig. S2C). We evaluated expression levels of the SFK family members Lck, Fyn, Hck, Lyn, and Fgr, as well as activity of the family using pan‐reactive anti‐pSFK Tyr416. In the IM‐resistant K562‐STI‐R cells, we found no difference in Src abundance. However, there was higher abundance of Lck, Fyn, and Hck. We also observed a marked increase in phospho‐SFK at the Tyr416 site (Fig. 3B, Fig. S2C). The pSrc Tyr527 signal was similar in the two cell lines. These data, in the aggregate, are consistent with involvement of SFKs in resistance to IM.

To confirm a direct effect of Tpl2 on activation of the MEK, NF‐κB, and SFK pathways, we overexpressed Tpl2 in the control K562 cells. The resulting data indicate that ectopic expression of Tpl2 leads to increased phosphorylation of Ser 271/221 on MEK1/2 as well as Thr 202/Tyr204 on ERK1/2. We also noted an increase in the phosphorylation status of Ser177/Ser181 on IKKα/β and Ser536 on p65 NF‐κB. The Tpl2‐overexpressing K562 cells also showed a modest increase in both the full‐length (p105a) and processed (p50) forms of the Tpl2‐binding partner NF‐κB1 (Fig. 3D). These data indicate that overexpression of Tpl2 positively affects MEK‐ERK and NF‐κB signaling. To determine the effect of the ectopic expression of Tpl2 on sensitivity of K562 cells to IM, we cultured Tpl2‐overexpressing K562 cells in the presence of 1, 10, and 25 μm of IM. The results indicate that overexpression of Tpl2 increases survival of Tpl2‐expressing K562 cells in the presence of the drug (Fig. 3E).

### 3.4 Combined inhibition of SFKs and MEK decreases survival of IM‐resistant cells

**Figure 4:**
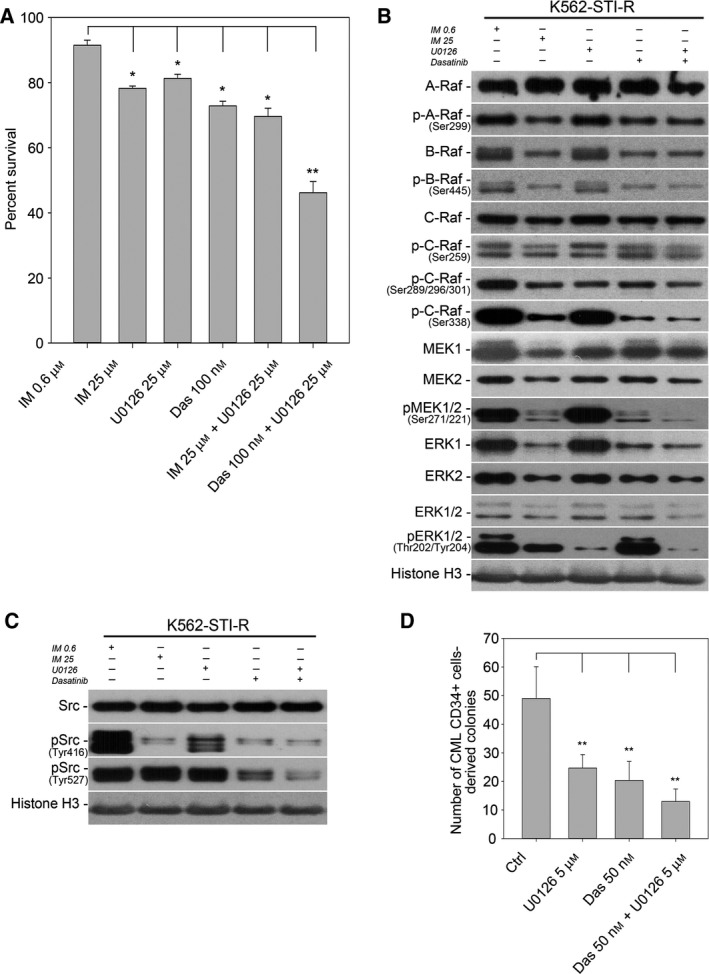
Sensitivity to SFK/MEK inhibition in IM‐resistant cells. (A) FACS analysis of the percentage of Annexin V‐FITC‐positive (apoptotic) cells in a population of K562 and K562‐STI‐R cells cultured with 25 μm IM, 25 μm U0126, 100 nm dasatinib, the combination of 25 μm IM and 25 μm U0126, and the combination of 100 nm dasatinib and 25 μm U0126. Data are shown as the mean and 1 SD for three independent analyses. *P < 0.05, **P < 0.01. Protein levels and phosphorylation status of (B) Raf‐MEK‐ERK pathway components (A‐, B‐, C‐Raf, MEK1 and 2, ERK 1 and 2) and (C) SFKs in K562‐STI‐R cells cultured with 25 μm IM, 25 μm U0126, 100 nm dasatinib, and the combination of 100 nm dasatinib and 25 μm U0126. Immunoblots representative of three independent experiments are shown. Quantification of the densitometry results for all three replicates, along with the results of statistical analyses, is summarized in Figures [Link], [Link], [Link]. (D) Number of colonies formed in the presence of U0126, dasatinib, or the combination of both. Colonies derived from burst‐forming units erythroid (BFU‐E), multilineage granulopoietic, erythroid, macrophage, and megakaryocytic colony‐forming units (CFU‐GEMM), granulocyte–macrophage colony‐forming units (CFU‐GM), and macrophage colony‐forming units (CFU‐M) were scored after 14 days incubation of CML CD34+ cells in methylcellulose‐based medium with cytokines in the presence or absence of 5 μm U0126, 50 nm dasatinib, and the combination of 5 μm U0126 and 50 nm dasatinib. This experiment was performed using CML CD34+ cells isolated prior to TKI exposure from the bone marrow of three CML patients. Data are shown as the mean and 1 SD. *P < 0.05, **P < 0.01.

Based on the upregulation of both SFK and MEK activity in IM‐resistant K562‐STI‐R cells, we investigated the effect of inhibiting of SFK and MEK signaling, individually and in combination, on the survival of IM‐resistant cells (Fig. 4A). The apoptotic effect of a potent Bcr‐Abl1 and SFKs inhibitor, dasatinib, and a MEK inhibitor, U0126, used individually and in combination, was compared to the effect of a high concentration of IM, 25 μm, previously shown by us to induce apoptosis in highly IM‐resistant K562‐STI‐R cells (Chorzalska *et al*., 2017). Dasatinib at 100 nm, 25 μm IM, or 25 μm U0126 all reduced survival of the IM‐resistant K562‐STI‐R cells by approximately 20–25% relative to control conditions, that is, K562‐STI‐R cells grown in basic medium containing 0.6 μm IM. The combination of 25 μm IM with 25 μm U0126 did not induce a further decrease in survival compared to exposure to the individual drugs. In contrast, the combination of 100 nm dasatinib with 25 μm U0126 resulted in a 49% decrease in survival. We interpreted these data as indicating a synergistic apoptosis‐inducing effect of SFK and MEK inhibition on IM‐resistant cells.

We next evaluated the effect of SFK and MEK inhibitors on SFK and MEK‐ERK signaling in the IM‐resistant cells (Fig. 4C; Figs [Link], [Link], [Link]). Treatment with high‐dose (25 μm) IM resulted in a decrease in phosphorylated MEK1/2 and ERK1/2, both of which could be partly accounted for by a reduction in total MEK1 and 2 and ERK1/2. B‐Raf and C‐Raf, but not A‐Raf content were also decreased. There was a marked reduction in the relative stoichiometry of A‐Raf phosphorylation. High‐dose IM exposure was also associated with a marked decrease in SFK Tyr416 phosphorylation that could not be accounted for by reduction in Src.

Exposure of K562‐STI‐R cells to 25 μm U0126 did not result in decreased MEK1 and 2 protein content or phosphorylation. Under these steady‐state conditions, ERK1 and ERK2 protein levels were also unaffected, however phosphorylation of ERK1/2 was significantly decreased, consistent with the known capacity of U0126 to inhibit activation of ERK1/2 by MEK1/2 (Favata *et al*., 1998) (Fig. 4B, Fig. S4). Treatment with 100 nm dasatinib resulted in changes in the Ras‐MEK‐ERK signaling module that were similar to those seen with 25 μm IM (Fig. 4B; Figs S3 and S4). Also, similar to high‐dose IM treatment was the lack of an effect of dasatinib on SFK content, while SFK phospho‐Tyr416 decreased (Fig. 4C, Fig. S3).

Finally, the combination of 100 nm dasatinib and 25 μm U0126 resulted in significant downregulation of the protein levels of MEK1 and 2 and ERK1 and 2 accompanied by a significant decrease in the phosphorylation status of both MEK1/2, ERK1/2 (Fig. 4B, Figs S3 and S4). The combination of SFK and MEK inhibitors also resulted in a decrease in SFK phospho‐Tyr416, while Src abundance remained unchanged (Fig. 4C, Fig. S5).

We assessed the direct effect of dasatinib, U0126, and the two inhibitors in combination on the capacity of CML CD34+ cells to form colonies in methylcellulose assays. The use of individual drugs significantly reduced the colony formation capacity by 50% in case of 5 μm U0126 and by 60% in case of 50 nm dasatinib. Surprisingly, the combination of 50 nm dasatinib and 5 μm U0126 showed only mild synergistic effect and resulted in a decrease of approximately 75% in the colony formation ability of CML CD34+ cells (Fig. 4D). Together, these data indicated that the effects of the SFKs and MEK inhibitors may only be in part additive. This, in turn, suggests that the two drugs may be acting through separate mechanisms or that the mechanistic overlap may be only partial and cell type dependent.

### 3.5 NF‐κB cascade inhibition potentiates the response to combined SFK and MEK inhibition in IM‐resistant cells

**Figure 5:**
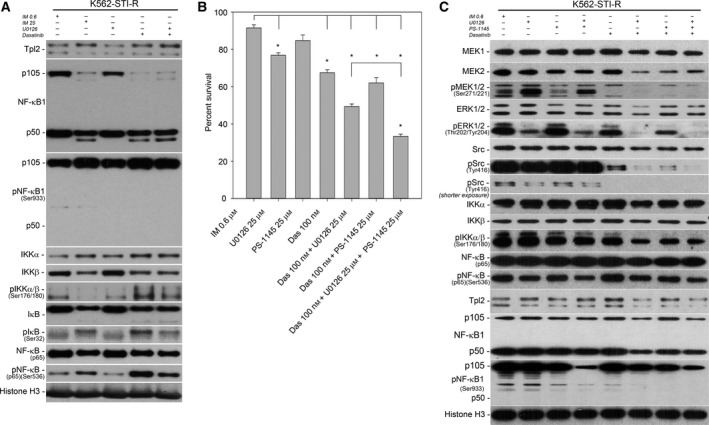
Sensitivity of IM‐resistant cells to SFK/MEK/IKK inhibition. (A) Western blot analysis was performed on K562 and K562‐STI‐R whole‐cell lysates. Protein levels and phosphorylation of NF‐κB pathway components (Tpl2, NF‐κB1 (p105, p50), IKK, IkB, and NF‐κB (p65)) were evaluated. Immunoblots representative of three independent experiments are shown. Histone H3 was used as a loading control. The densitometry results of the western blots along with statistical analyses are summarized in Fig. S6B. (B) FACS analysis of the percentage of Annexin V‐FITC‐positive (apoptotic) cells in a population of K562 and K562‐STI‐R cells incubated with 25 μm U0126, 25 μm PS‐1145, 100 nm dasatinib, the combination of 100 nm dasatinib and 25 μm U0126, the combination of 100 nm dasatinib and 25 μm PS‐1145, or the combination of 100 nm dasatinib, 25 μm U0126, and 25 μm PS‐1145. Data are shown as the mean and 1 SD for three independent analyses, *P < 0.05. (C) Protein levels and phosphorylation status of MEK1 and 2, ERK 1 and 2, SFKs, IKK, and NF‐κB (p65) in K562‐STI‐R cells incubated with 25 μm U0126, 25 μm PS‐1145, 100 nm dasatinib, the combination of 25 μm U0126 and 25 μm PS‐1145, the combination of 100 nm dasatinib and 25 μm U0126, the combination of 100 nm dasatinib and 25 μm PS‐1145, or the combination of 100 nm dasatinib, 25 μm PS‐1145 and 25 μm U0126. Representative immunoblots of three independent experiments are shown. The densitometry results of the western blots along with statistical analyses are summarized in Figs S3–S5.

We next evaluated the effect of combined SFK and MEK inhibition on Tpl2‐NF‐κB signaling. Treatment with IM, dasatinib, or the combination of dasatinib and U0126 did not alter Tpl2 levels (Fig. 5A). However, we did observe a decrease in p105 NF‐κB1 levels, most likely due to induction of its proteolytic degradation by IM, dasatinib, or the combination of dasatinib and U0126 treatments. Consistent with this was an increase in the phosphorylation of NF‐κB1 at Ser933 in cells treated with high‐dose IM, dasatinib, or dasatinib plus U0126 (Fig. 5A). These treatments also resulted in an increase in IKKα and a decrease in IKKβ protein levels. U0126 or 25 μM IM alone induced a decrease in IKKαβ phosphorylation on Ser 176/180. However, dasatinib and combination of dasatinib and U0126 resulted in an increase in IKKαβ phosphorylation on Ser 176/180 (Fig. 5A). An increase in the phosphorylation status of IkB on Ser32 and NF‐κB p65 on Ser536 was observed in K562‐STI‐R cells treated with high‐dose IM, dasatinib, or the combination of dasatinib and U0126 (Fig. 5A).

These data indicated that while dual inhibition of SFKs and MEK resulted in suppression of the activity of these pathways, it did not induce an inhibitory effect on NF‐κB signaling. We therefore determined whether addition of the IKK inhibitor PS‐1145 would potentiate the effects of SFK/MEK inhibition on survival of IM‐resistant cells (Fig. 5B). IKK inhibition alone did not induce a significant apoptotic effect. In combination with 100 nm dasatinib, it induced only a relatively modest 25% decrease in survival of IM‐resistant cells. However, the combination of dasatinib, U0126, and PS‐1145 induced a 65% decrease in survival of IM‐resistant cells, surpassing the effect induced by the combination of dasatinib and U0126 (Fig. 5B).

PS‐1145 alone or in combination with U0126 had no discernible effect on SFK, MEK‐ERK, and NF‐κB signaling in IM‐resistant cells (Fig. 5C, Figs S3 and S4). However, when the IKK inhibitor was used in combination with U0126 and dasatinib, it induced a significant decrease in phosphorylation of MEK1/2, ERK1/2, SFKs, and NF‐κB that exceeded the effect of combined dasatinib and U0126 (Fig. 5C, Figs S3, S4, and S6).

**Figure 6:**
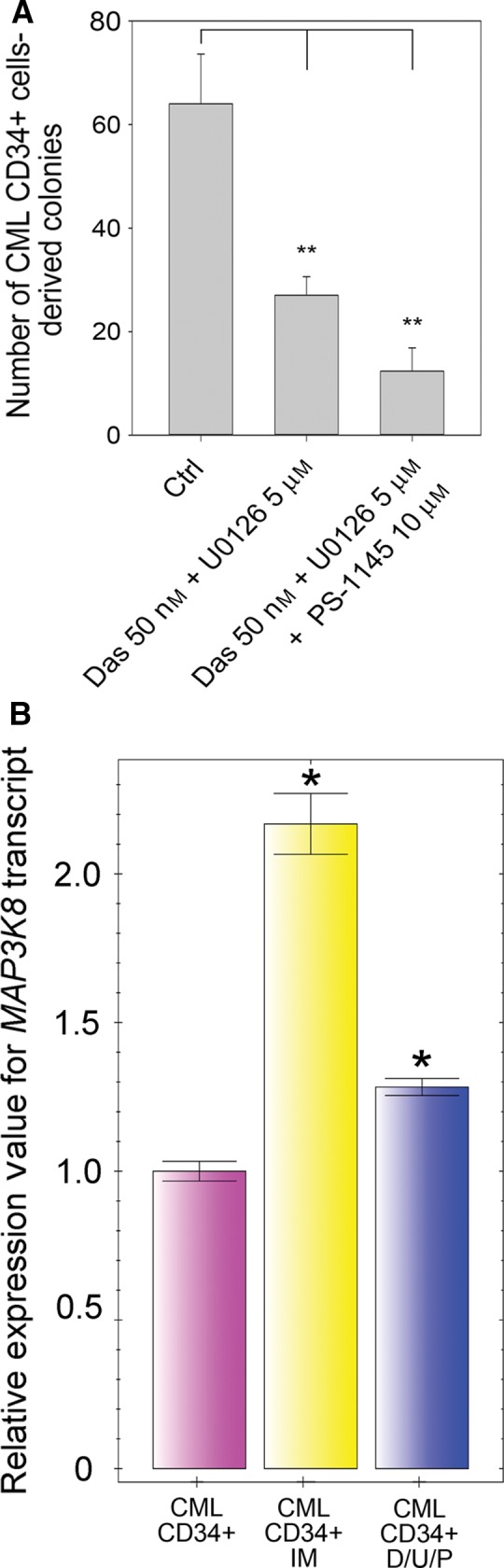
Sensitivity of CML CD34+ cells to SFK/MEK/IKK inhibition. (A) Number of colonies formed in the absence or presence of the combination of dasatinib and U0126, or the combination of dasatinib, U0126, and PS‐1145. Colonies derived from burst‐forming units erythroid (BFU‐E), multilineage granulopoietic, erythroid, macrophage, and megakaryocytic colony‐forming units (CFU‐GEMM), granulocyte–macrophage colony‐forming units (CFU‐GM), and macrophage colony‐forming units (CFU‐M) were scored *in situ* after 14 days of incubation of CML CD34+ cells in methylcellulose‐based medium with cytokines in the presence or absence of the aforementioned combinations of inhibitors. This experiment was performed using CML CD34+ cells isolated prior to TKI exposure from the bone marrow of three CML patients. Data are shown as the mean and 1 SD. **P < 0.01. (B) RT‐PCR determination of *MAP3K8* transcript levels in CML CD34+ cells cultured without inhibitors or with a combination of 50 nm dasatinib, 5 μm U0126, and 10 μm PS‐1145 (D/U/P) or with IM for 7 days. Normalized expression relative to *ACTB* and *S18* rRNA expressed as fold‐change is presented. Samples were cultured and analyzed in triplicate. Results are shown as the mean and 1 SD. *P < 0.05.

We also compared the effect of dasatinib plus U0126 with the combination of dasatinib, U0126, and PS‐1145 on the capacity of CML CD34+ cells to form colonies in methylcellulose assays (Fig. 6A). The combination of dasatinib and U0126 resulted in a 62% reduction in colony number. In contrast, the combination of dasatinib, U016, and PS‐1145 resulted in 80% reduction in colony formation number. Finally, CML CD34+ cells cultured for 7 days in the presence of SFKs, MEK, and IKK inhibitors showed a normalizing effect on *MAP3K8* transcript levels (Fig. 6B). We interpreted these data as indicating that the combination of SFK, MEK, and NF‐κB inhibitors limits the survival of primary CML cells displaying Bcr‐Abl1‐independent mechanisms of survival.

## 4 Discussion

Tpl2 is a member of the serine/threonine mitogen‐activated protein kinase (MAPK) kinase kinase (MAP3K) family. Members of this kinase family propagate cytokine, growth factor, stress, and radiation‐initiated signals. They elicit an array of responses affecting inflammation, proliferation, survival, apoptosis, migration, and differentiation. There is a lack of consensus regarding the role of Tpl2 in the development and progression of cancer. Its tumor suppressor and oncogenic roles have both been documented (reviewed in Lee *et al*., 2015). These contradictory effects of Tpl2 most likely originate from the cancer type‐specific extrinsic (microenvironment‐dependent) and intrinsic (signaling) events involving this kinase. The dual role of Tpl2 and its cancer type dependence are also evident from its expression patterns in The Cancer Genome Atlas (TCGA) datasets where downregulation of *MAP3K8* occurs in some cancers and upregulation occurs in others (Table S3). More specifically, overexpression of Tpl2 has been shown to drive resistance to Raf inhibition in melanoma (Johannessen *et al*., 2010; Monsma *et al*., 2015); however, the role of elevated Tpl2 protein levels in resistance to IM or, more generally, drug resistance in leukemia, has not been explored. To our knowledge, this is the first report indicating involvement of Tpl2 in resistance to TKIs.

**Figure 7:**
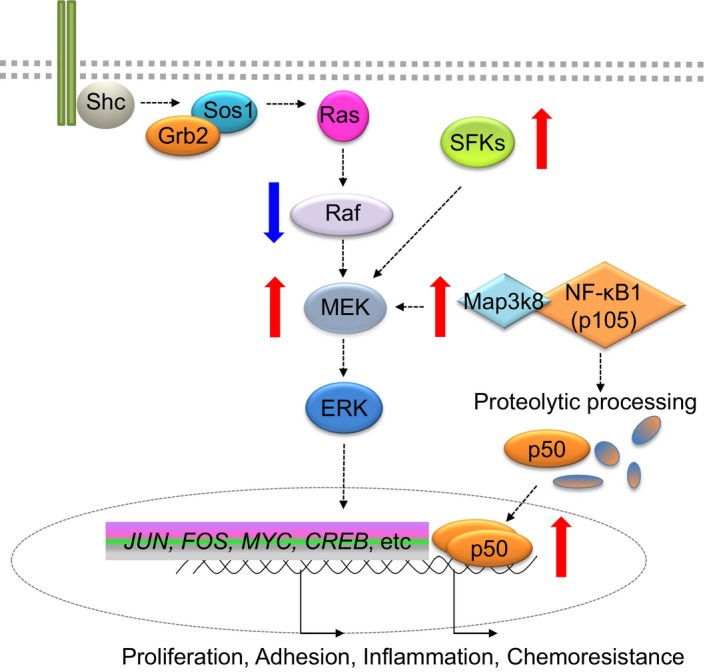
Proposed model of SFK, MEK‐ERK, and NF‐κB signaling cross‐talk in CML stem cells insensitive to imatinib mesylate. It is proposed that while TKI treatment deactivates RAF module it leads to overexpression/overactivity of SFK and Tpl‐2 leading to MEK hyperactivity, proteolytic processing of NF‐κB1 and increase in its transcriptional activity. Red arrows indicate overexpression/overactivity, blue arrows indicate downregulation.

We embarked on an investigation of mechanisms for IM resistance in CML by performing a phosphoproteomic analysis of corresponding IM‐sensitive and IM‐resistant cells. Given the role of Tpl2 in activation of both MEK‐ERK and NF‐κB signaling, and the upregulation of these two pathways in chemoresistant leukemic stem cells (Guzman *et al*., 2001; Steelman *et al*., 2011b), we paid particular attention to results pointing to a role for Tpl2 in IM resistance. To explore the potential clinical relevance of our initial observations, we determined transcript levels of both *MAP3K8* and its binding partner *NFKB1* in CML CD34+ cells exposed to IM. We observed a significant increase in the transcript levels of both genes in the resistant cells, suggesting their possible role in intrinsic, Bcr‐Abl1‐independent insensitivity of CML stem cells to IM. Because we observed an increased abundance of Tpl2 phosphopeptides in our cellular model of IM resistance together with elevated protein levels of Tpl2, and knowing that Tpl2 is a molecule able to control activation of both MEK‐ERK and NF‐κB cascades, we determined their activity status in IM‐resistant cells. Evaluation of the MEK‐ERK signaling activation in our cellular model of IM resistance by quantitative phosphoproteomics and immunoblotting indicated elevated levels of phosphorylation of MEK1/2, which was independent of apparent Raf activation. To determine upstream factors contributing to the activation of MEK, we evaluated the activity status of SFKs, which have been shown to be activated in imatinib and dasatinib‐resistant CML patient samples (Hayette *et al*., 2011). Indeed, activation of SFKs, detected by the SFK pan‐specific Tyr416 antibody, was observed in IM‐resistant cells. Our phosphoproteomic and immunoblotting results led us to formulate a model of signaling cross‐talk in IM‐insensitive CML stem cells (Fig. 7). Upregulated SFK and MEK activity served as a rationale to test the effect of dual SFKs/MEK inhibition on the survival of an IM‐resistant cell line and IM‐insensitive CML CD34+ cells. For these tests, we chose the combination of dasatinib, a potent SFKs inhibitor with IC_50_ < 1 nm (Lombardo *et al*., 2004; O'Hare *et al*., 2005) and U0126 (also known as PD184352), a potent MEK1/2 inhibitor with IC_50_ of 0.07 μm (Duncia *et al*., 1998). This combination previously showed a strong pro‐apoptotic effect on CML cell lines that were resistant to IM due to (i) mutations in Bcr‐Abl1 kinase domain, (ii) overexpression of Bcr‐Abl1, or (iii) overexpression of Lyn (Nguyen *et al*., 2007). This combination had not been tested in cellular models of Bcr‐Abl1‐independent resistance to IM. In our model, the combination of 100 nm dasatinib with 25 μm U0126 showed a significant reduction in survival of IM‐resistant cells, as well as significant reduction in the ability of CML CD34+ cells to form colonies in methylcellulose assays. Our data indicate that the effects of the SFKs and MEK inhibitors are only in part additive, suggesting that they may be acting through separate mechanisms or that the mechanistic overlap between the pathways may be only partial, cell type dependent and affected by activation of other signaling cascades.

We also evaluated the effect of SFKs/MEK inhibition on the NF‐κB signaling module. We did not observe significant changes within the IKK/NF‐κB activity status in IM‐resistant cells exposed to SFKs/MEK inhibition. Our observations led us to conclude that even though combined SFKs/MEK inhibition was able to significantly reduce the number of IM‐resistant cells, IKK/NF‐κB signaling remained unaffected by the combination of dasatinib and U0126 and may be responsible for the survival of chemoresistant cells. We therefore decided to test the effect of combined inhibition of SFKs, MEK, and NF‐κB cascades on the survival of IM‐resistant cells. Because the hyperactivity of NF‐κB is considered one of the ‘hallmarks’ of cancer, and its role in chemoresistance is gaining recognition, substantial efforts within the recent decade have been made toward development of NF‐κB inhibitors (DiDonato *et al*., 2012; Gilmore and Herscovitch, 2006). This has proved challenging due to the ubiquitous nature of NF‐κB signaling, its pleiotropic physiological functions, the structural characteristics of the NF‐κB family of transcription factors, and complexity of the NF‐κB signaling network. Several groups of NF‐κB pathway inhibitors have been developed. These include inhibitors of NF‐κB protein kinases (mainly IKK), proteasome inhibitors, IκB ubiquitination blockers, NF‐κB p65 acetylation blockers, inhibitors of NF‐κB nuclear translocation, and DNA binding. While testing of all of these agents is warranted, we focused on the IKKβ inhibitor, PS‐1145. Our choice was influenced by the reported observation that PS‐1145 alone is able to induce apoptotic response in IM‐resistant CML cells (Cilloni *et al*., 2006). Our analyses indicated that a combination of inhibitors targeting SFKs, MEK, and NF‐κB pathways can surpass the efficacy of SFK/MEK dual inhibition in reducing the number of IM‐resistant cells and of the CML CD34+‐initiated colonies.

One of our observations that is difficult to interpret is the dissociation of MEK inhibition from an effect on ERK activity. Indeed, while MEK‐independent activation of ERK is widely accepted (Levin‐Salomon *et al*., 2008; Simard *et al*., 2015), uncoupling MEK downstream signaling to ERK remains controversial. However, activation of a MEK without the corresponding activation of the MEK substrate ERK has been already reported (Boylan and Gruppuso, 1998; Harding *et al*., 2003). In addition, recent studies indicating that ERK may not be the only substrate of MEK (Tang *et al*., 2015) challenge the prevailing paradigm that ERK exclusively instigates the effects of RAS‐RAF‐MEK signaling. In light of these findings, it may seem reasonable to revisit the issue of ERK‐independent effects of MEK activation.

While dysregulation in signaling by the PI3K/AKT/FOXO, Hedgehog, Wnt and Jak/STAT pathways, as well as DNA damage repair regulating pathways, has been putatively involved in CML stem cells insensitivity to treatment, involvement of the NF‐κB signaling, particularly in conjunction with increased activities of SFKs and MEK‐ERK, in the resistance of CML stem cells to TKIs has not been previously identified. While detailed studies are required to determine the optimal composition of available SFKs, MEK, and NF‐κB inhibitors to ensure maximal eradication of chemotherapy‐unresponsive stem cells, our report indicates that the pursuit of such strategy is reasonable.

## Author contributions

PMD planned and designed the research, performed and analyzed experiments, and wrote the manuscript. AC, AT, SH performed and analyzed experiments and contributed to writing. NA, RSPR performed phosphoproteomic experiments and subsequent analyses and contributed to writing. XY, JM, PZ, DOT, TCZ, AJO, JLR, OL, PAG helped analyze and interpret the data and contributed to writing.

## Supporting information


**Fig. S1.** (A) Detailed map of the bicistronic pENTR‐CMV‐HBG‐3xHA‐IRES‐hrGFP vector. This vector allows the CMV promoter‐driven co‐expression of an open‐reading frame cloned between unique BamHI and XhoI sites and a humanized *Renilla reniformis* GFP via an IRES. The upstream human beta globin intron serves to increase transcription. (B) Histograms showing K562 cells electroporated with control or Tpl2 p58‐encoding vectors. Presented data are from three independent electroporation experiments, GFP positive K562 cells were sorted.

**Fig. S2.** Densitometric analysis of the expression of MEK1, MEK2 and phospho‐MEK1/2, (B) ERK1, ERK2 and phospho‐ERK1/2 and (C) NF‐κB and phospho‐NF‐κB in K562 and K562‐STI‐R cells. The intensities of bands are expressed as the relative intensity fold‐change, with the intensity of each band normalized to control (K562) cells. Whole cell lysates of K562 and their IM‐resistant counterpart (K562‐STI‐R) cells were collected in four independent experiments. Immunoblotting and densitometry analyses were performed on four sample sets using antibodies detecting (A) MEK1 #2352, MEK2 #9125, phospho‐MEK1/2 (Ser 217/221) #9154, (B) both ERK1 and ERK2: p44/42 MAPK (Erk1/2) #9102 and phospho‐ERK1 and ERK2: phospho‐p44/42 MAPK (Erk1/2) (Thr202/Tyr204) (D13.14.4E) XP #4370 and (C) Src #2123 and phospho‐Src (Tyr 416) #6943. Histone H3 was used as a loading control. *P < 0.05.

**Fig. S3.** Densitometric analysis of the expression of MEK1, MEK2 and phospho‐MEK1/2 in K562‐STI‐R cells (untreated) and K562‐STI‐R cells cultured in the presence of 25 μm IM, 25 μm U0126, 25 μm PS‐1145, 100 nm dasatinib and combinations of 100 nm dasatinib and 25 μm PS‐1145, 25 μm U0126 and 25 μm PS‐1145, 100 nm dasatinib and 25 μm U0126 and 100 nm dasatinib, 25 μm U0126 and 25 μm PS‐1145.

**Fig. S4.** Densitometric analysis of the expression of ERK1/2 and phospho‐ERK1/2 in K562‐STI‐R cells (untreated) and K562‐STI‐R cells cultured in the presence of 25 μm IM, 25 μm U0126, 25 μm PS‐1145, 100 nm dasatinib, the combination of 100 nm dasatinib and 25 μm PS‐1145, the combination of 25 μm U0126 and 25 μm PS‐1145, the combination of 100 nm dasatinib and 25 μm U0126, or the combination of 100 nm dasatinib, 25 μm U0126 and 25 μm PS‐1145.

**Fig. S5.** Densitometric analysis of the expression of Src and phospho‐Src in K562‐STI‐R cells (untreated) and K562‐STI‐R cells cultured in the presence of 25 μm IM, 25 μm U0126, 25 μm PS‐1145, 100 nm dasatinib, the combination of 100 nm dasatinib and 25 μm PS‐1145, the combination of 25 μm U0126 and 25 μm PS‐1145, the combination of 100 nm dasatinib and 25 μm U0126, or the combination of 100 nm dasatinib, 25 μm U0126 and 25 μm PS‐1145.

**Fig. S6.** Densitometric analysis of the expression of NF‐κB and phospho‐NF‐κB in (A) K562 and K562‐STI‐R cells and (B) in K562‐STI‐R cells (untreated) and K562‐STI‐R cells cultured in the presence of 25 μm IM, 25 μm U0126, 25 μm PS‐1145, 100 nm dasatinib and combinations of 100 nm dasatinib and 25 μm PS‐1145, 25 μm U0126 and 25 μm PS‐1145, 100 nm dasatinib and 25 μm U0126 and 100 nm dasatinib, 25 μm U0126 and 25 μm PS‐1145.

**Table S1.** Comparison of abundances of phosphopeptides identified in K562 vs. K562‐STI‐R cells by quantitative phosphoproteomics.

**Table S2.** Comparison of the abundances of MAPK‐ERK superpath related phosphopeptides in K562 vs. K562‐STI‐R cells.

**Table S3.** Tpl2 expression patterns in various cancers based on the data from The Cancer Genome Atlas (TCGA) datasets.
