# Supplementary figures and images for: Overexpression of Tpl2 is linked to imatinib resistance and activation of MEK‐ERK and NF‐κB pathways in a model of chronic myeloid leukemia
Source: Mol Oncol. 2018 Apr 6;12(5):630–47. doi: 10.1002/1878-0261.12186 (PMC5928369; doi:10.1002/1878-0261.12186)

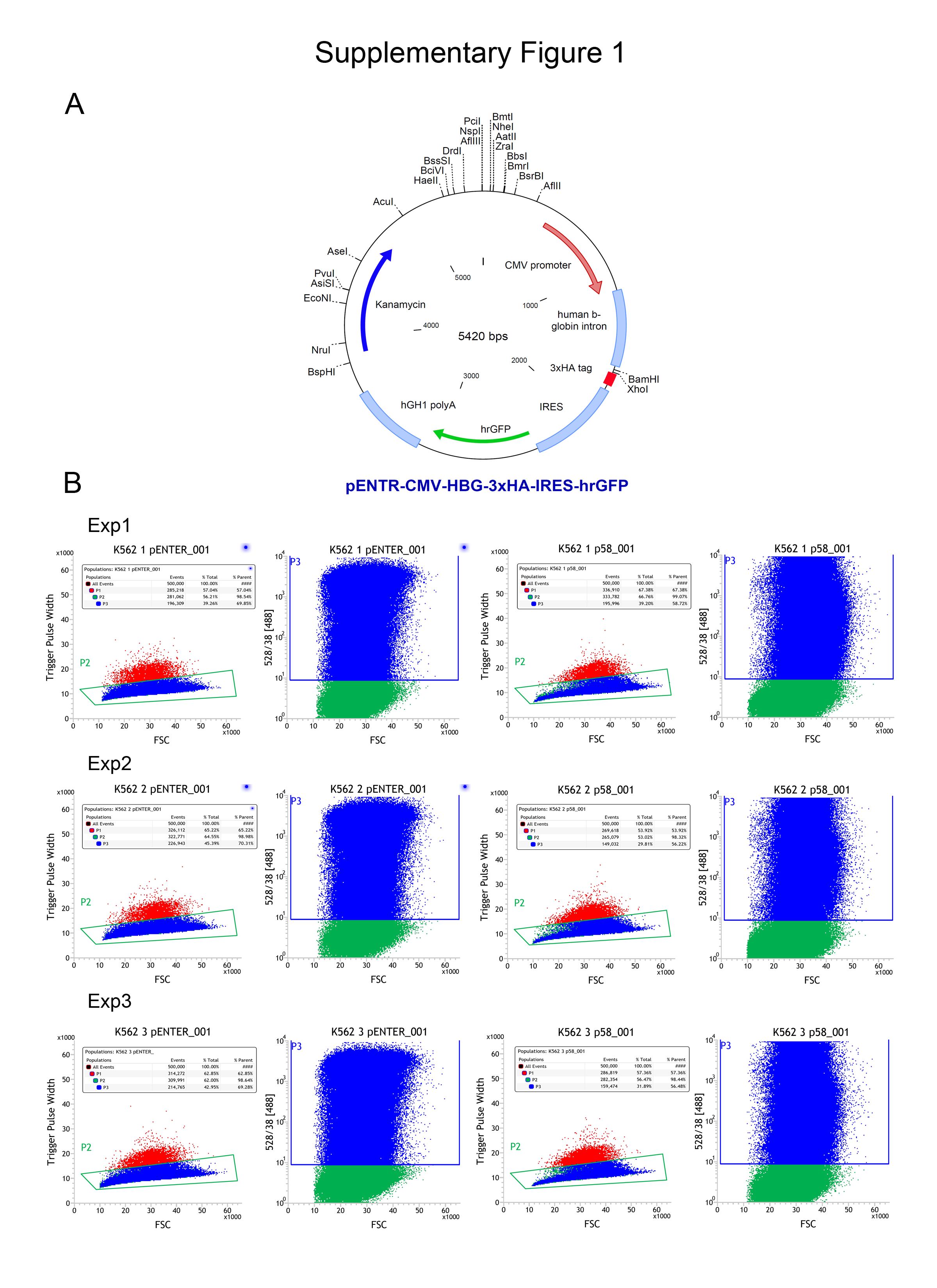

Supplement: Supplementary file 1 — Fig. S1. (A) Detailed map of the bicistronic pENTR‐CMV‐HBG‐3xHA‐IRES‐hrGFP vector. This vector allows the CMV promoter‐driven co‐expression of an open‐reading frame cloned between unique BamHI and XhoI sites and a humanized Renilla reniformis GFP via an IRES. The upstream human beta globin intron serves to increase transcription. (B) Histograms showing K562 cells electroporated with control or Tpl2 p58‐encoding vectors. Presented data are from three independent electroporation experiments, GFP positive K562 cells were sorted. [file MOL2-12-630-s001.tif]

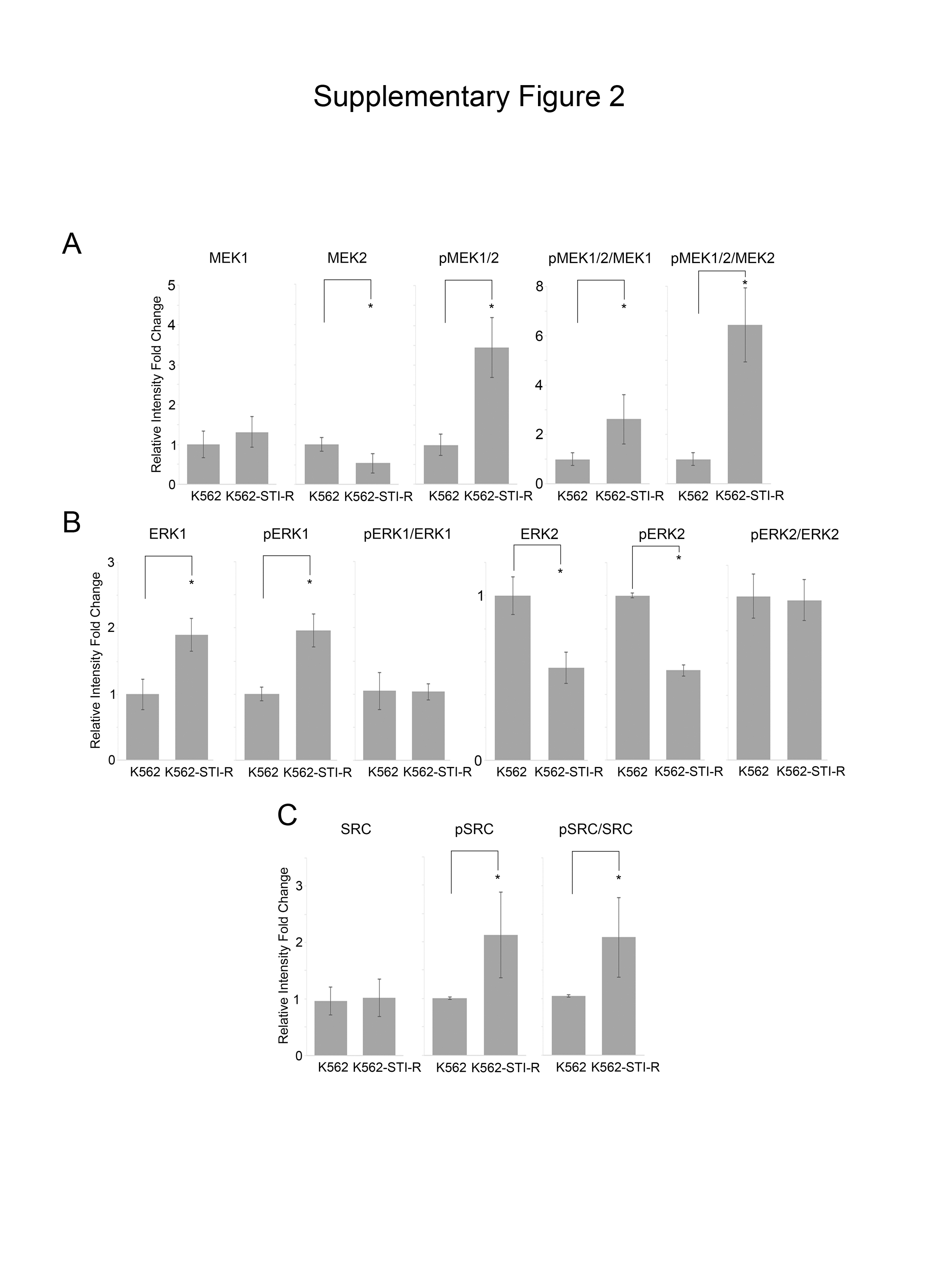

Supplement: Supplementary file 2 — Fig. S2. Densitometric analysis of the expression of MEK1, MEK2 and phospho‐MEK1/2, (B) ERK1, ERK2 and phospho‐ERK1/2 and (C) NF‐κB and phospho‐NF‐κB in K562 and K562‐STI‐R cells. The intensities of bands are expressed as the relative intensity fold‐change, with the intensity of each band normalized to control (K562) cells. Whole cell lysates of K562 and their IM‐resistant counterpart (K562‐STI‐R) cells were collected in four independent experiments. Immunoblotting and densitometry analyses were performed on four sample sets using antibodies detecting (A) MEK1 #2352, MEK2 #9125, phospho‐MEK1/2 (Ser 217/221) #9154, (B) both ERK1 and ERK2: p44/42 MAPK (Erk1/2) #9102 and phospho‐ERK1 and ERK2: phospho‐p44/42 MAPK (Erk1/2) (Thr202/Tyr204) (D13.14.4E) XP #4370 and (C) Src #2123 and phospho‐Src (Tyr 416) #6943. Histone H3 was used as a loading control. *P < 0.05. [file MOL2-12-630-s002.tif]

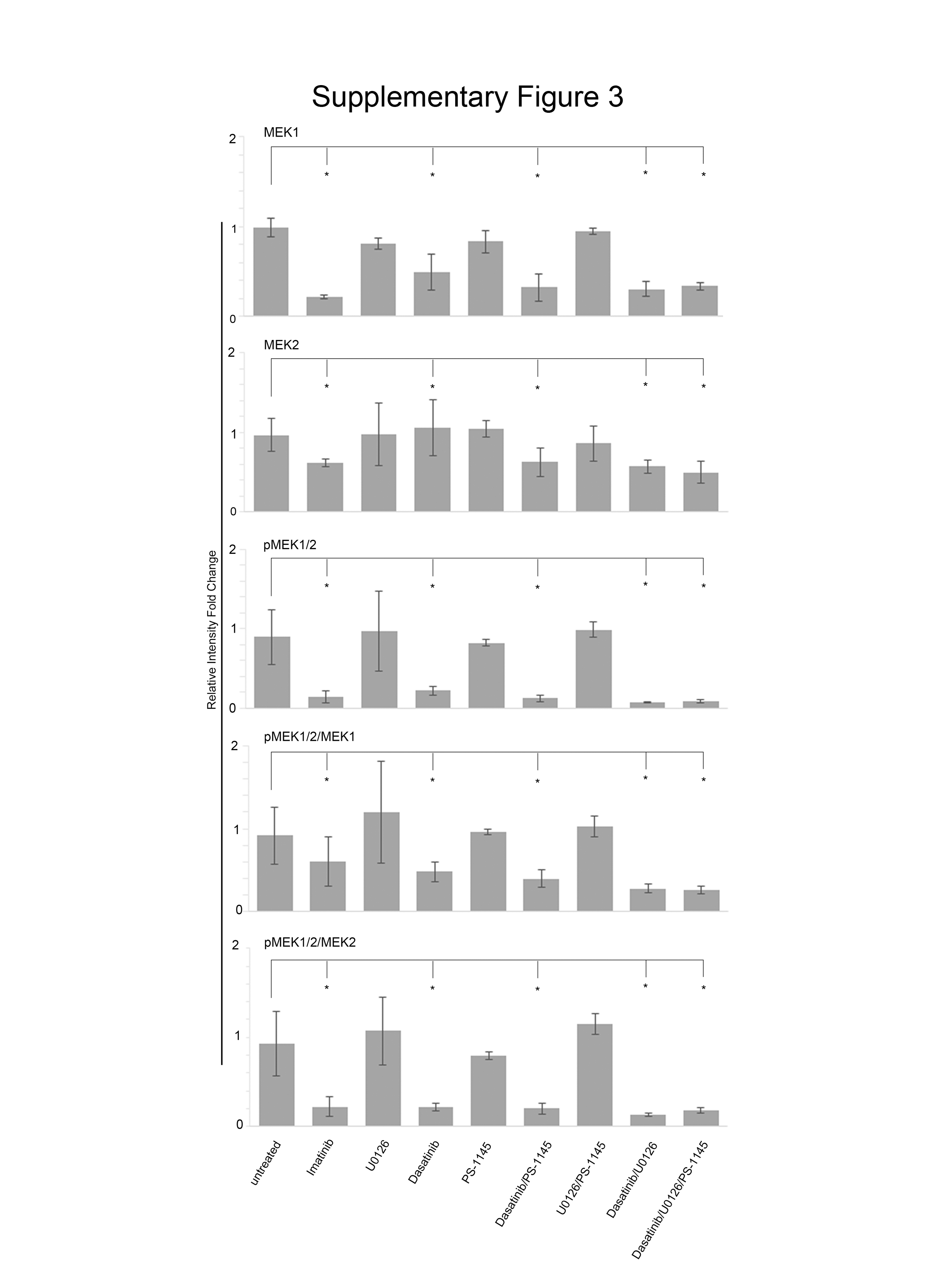

Supplement: Supplementary file 3 — Fig. S3. Densitometric analysis of the expression of MEK1, MEK2 and phospho‐MEK1/2 in K562‐STI‐R cells (untreated) and K562‐STI‐R cells cultured in the presence of 25 μm IM, 25 μm U0126, 25 μm PS‐1145, 100 nm dasatinib and combinations of 100 nm dasatinib and 25 μm PS‐1145, 25 μm U0126 and 25 μm PS‐1145, 100 nm dasatinib and 25 μm U0126 and 100 nm dasatinib, 25 μm U0126 and 25 μm PS‐1145. [file MOL2-12-630-s003.tif]

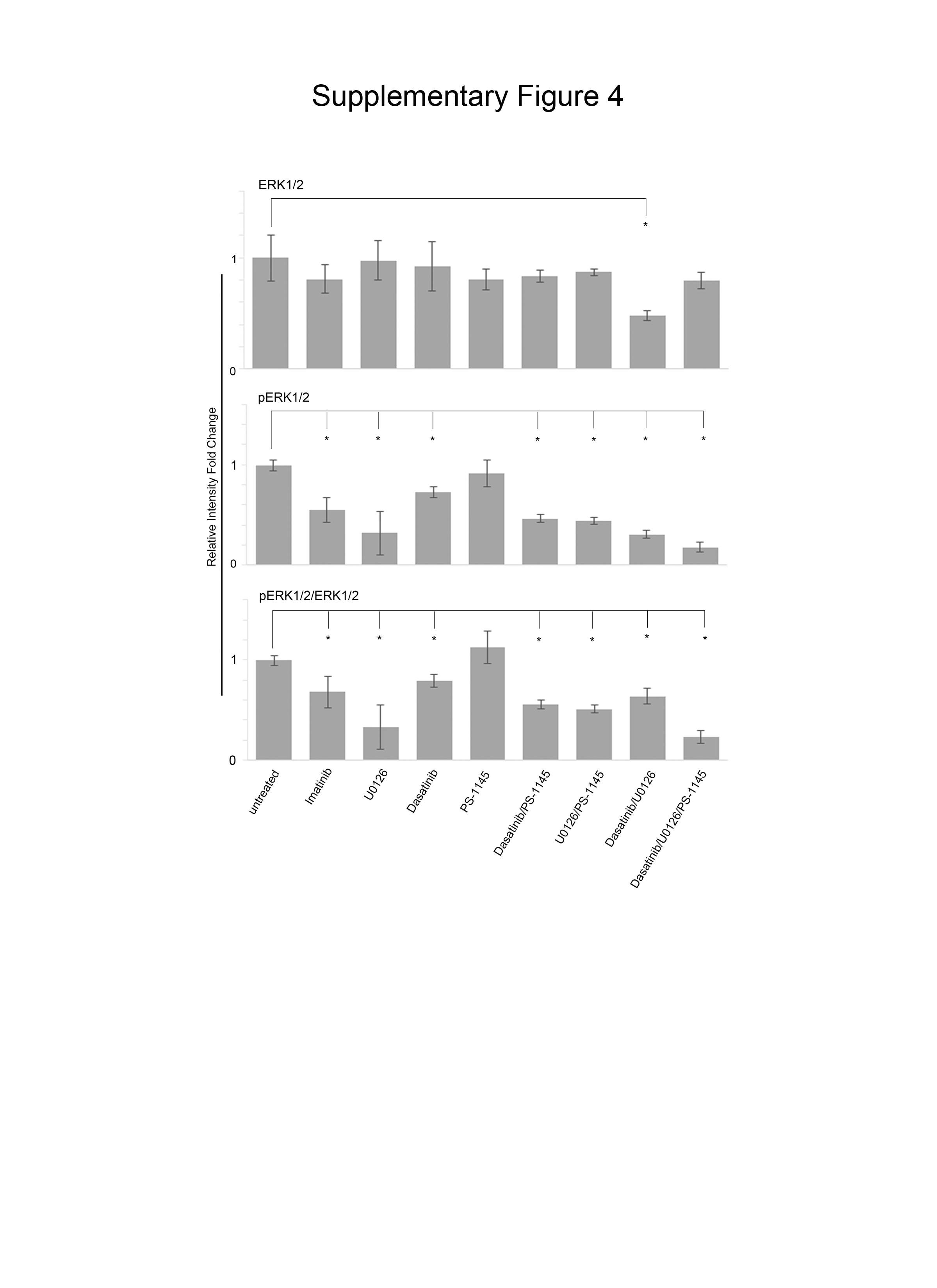

Supplement: Supplementary file 4 — Fig. S4. Densitometric analysis of the expression of ERK1/2 and phospho‐ERK1/2 in K562‐STI‐R cells (untreated) and K562‐STI‐R cells cultured in the presence of 25 μm IM, 25 μm U0126, 25 μm PS‐1145, 100 nm dasatinib, the combination of 100 nm dasatinib and 25 μm PS‐1145, the combination of 25 μm U0126 and 25 μm PS‐1145, the combination of 100 nm dasatinib and 25 μm U0126, or the combination of 100 nm dasatinib, 25 μm U0126 and 25 μm PS‐1145. [file MOL2-12-630-s004.tif]

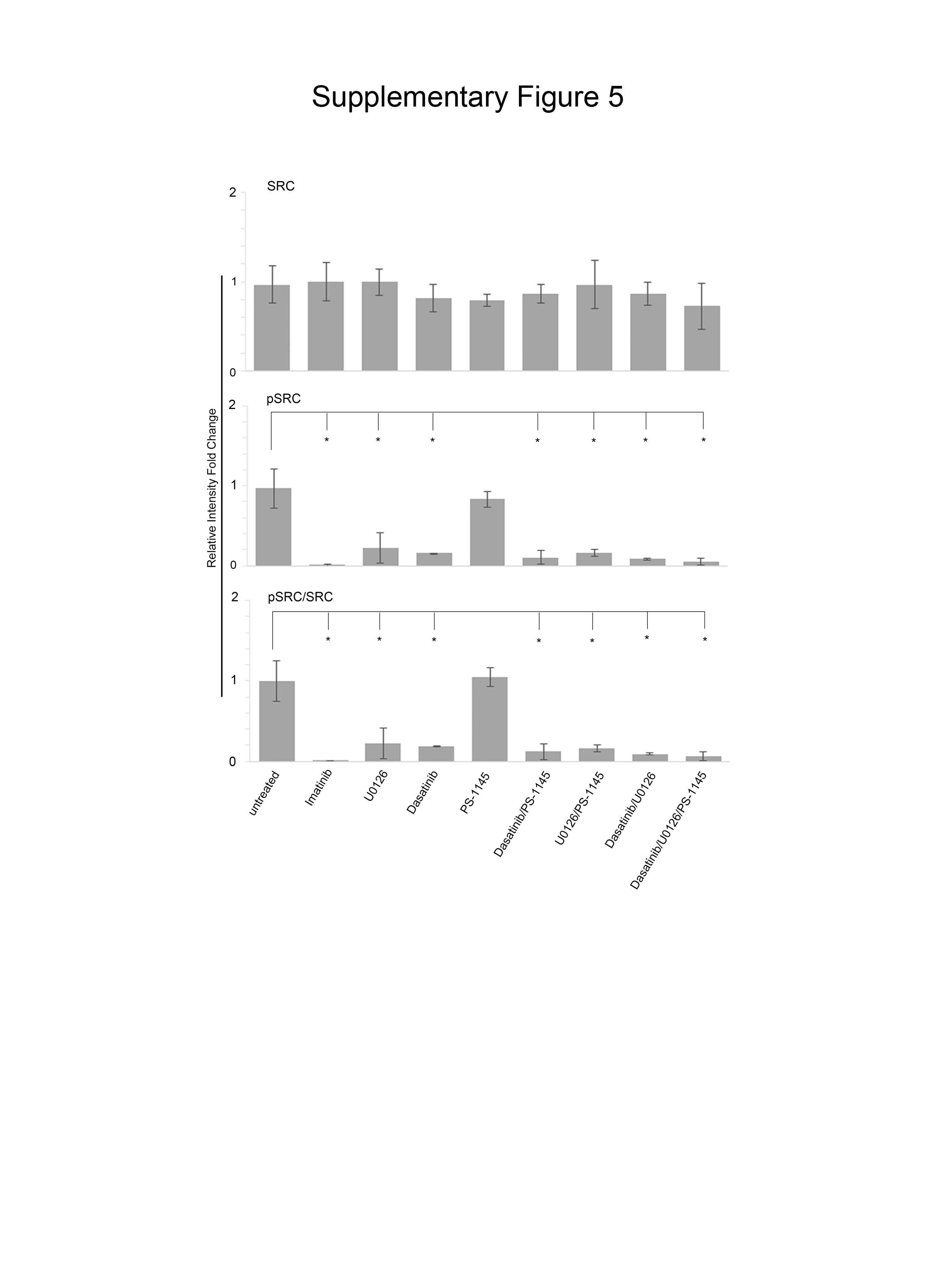

Supplement: Supplementary file 5 — Fig. S5. Densitometric analysis of the expression of Src and phospho‐Src in K562‐STI‐R cells (untreated) and K562‐STI‐R cells cultured in the presence of 25 μm IM, 25 μm U0126, 25 μm PS‐1145, 100 nm dasatinib, the combination of 100 nm dasatinib and 25 μm PS‐1145, the combination of 25 μm U0126 and 25 μm PS‐1145, the combination of 100 nm dasatinib and 25 μm U0126, or the combination of 100 nm dasatinib, 25 μm U0126 and 25 μm PS‐1145. [file MOL2-12-630-s005.tif]

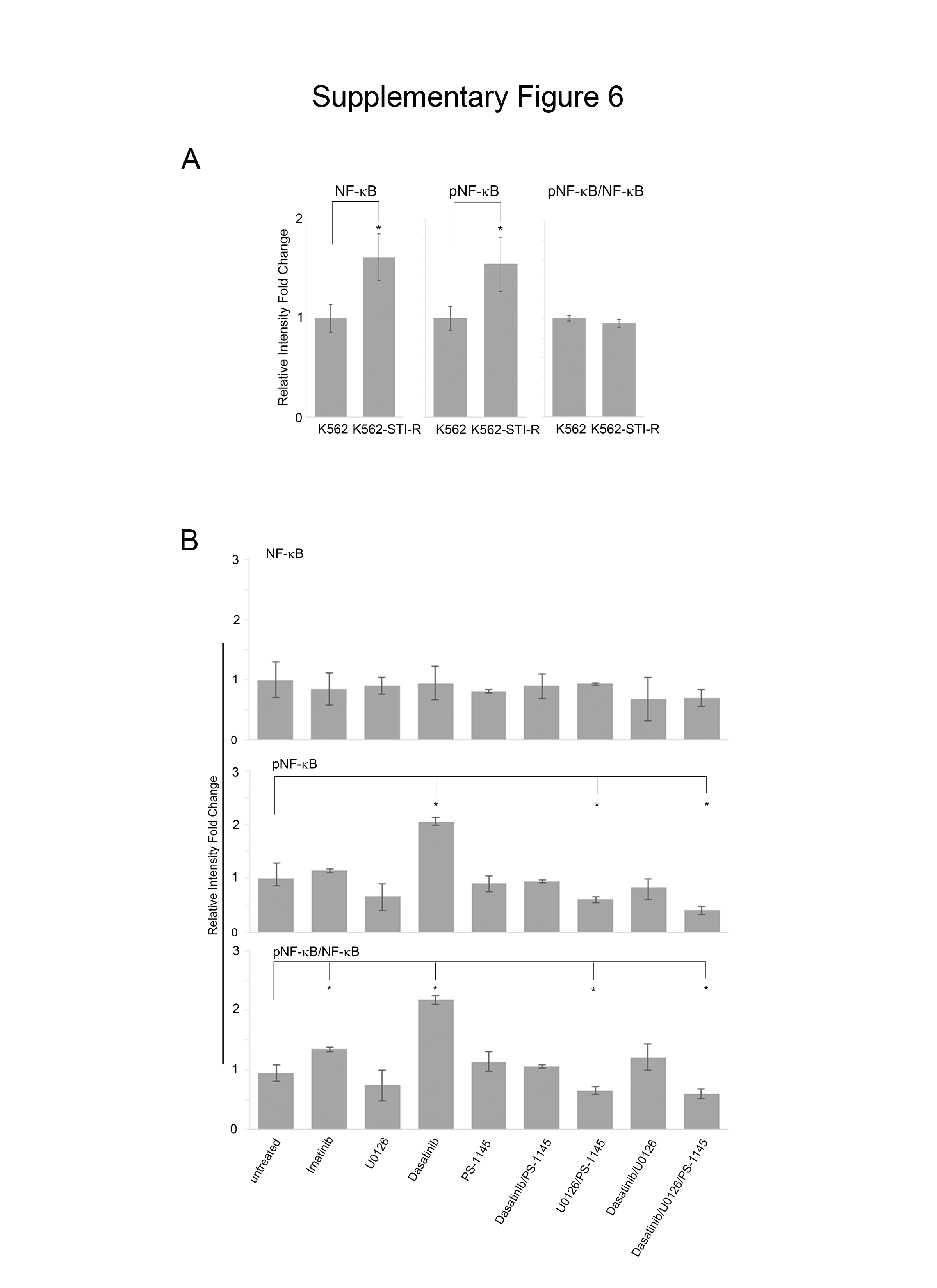

Supplement: Supplementary file 6 — Fig. S6. Densitometric analysis of the expression of NF‐κB and phospho‐NF‐κB in (A) K562 and K562‐STI‐R cells and (B) in K562‐STI‐R cells (untreated) and K562‐STI‐R cells cultured in the presence of 25 μm IM, 25 μm U0126, 25 μm PS‐1145, 100 nm dasatinib and combinations of 100 nm dasatinib and 25 μm PS‐1145, 25 μm U0126 and 25 μm PS‐1145, 100 nm dasatinib and 25 μm U0126 and 100 nm dasatinib, 25 μm U0126 and 25 μm PS‐1145. [file MOL2-12-630-s006.tif]
