# Supplementary material for: Overexpression of Tpl2 is linked to imatinib resistance and activation of MEK‐ERK and NF‐κB pathways in a model of chronic myeloid leukemia
Source: Mol Oncol. 2018 Apr 6;12(5):630–47. doi: 10.1002/1878-0261.12186 (PMC5928369; doi:10.1002/1878-0261.12186)
Supplement: Supplementary file 10 [file MOL2-12-630-s010.docx]

Fig. S1. (A) Detailed map of the bicistronic pENTRCMV- HBG-3xHA-IRES-hrGFP vector. This vector allows the CMV promoter-driven co-expression of an open-reading frame cloned between unique BamHI and XhoI sites and a humanized *Renilla reniformis* GFP via an IRES. The upstream human beta globin intron serves to increase transcription. (B) Histograms showing K562 cells electroporated with control or Tpl2 p58-encoding vectors. Presented data are from three independent electroporation experiments, GFP positive K562 cells were sorted.

Fig. S2. Densitometric analysis of the expression of MEK1, MEK2 and phospho-MEK1/2, (B) ERK1, ERK2 and phospho-ERK1/2 and (C) NF-jB and phospho-NF-jB in K562 and K562-STI-R cells. The intensities of bands are expressed as the relative intensity fold-change, with the intensity of each band normalized to control (K562) cells. Whole cell lysates of K562 and their IM-resistant counterpart (K562-STI-R) cells were collected in four independent experiments. Immunoblotting and densitometry analyses were performed on four sample sets using antibodies detecting (A) MEK1 #2352, MEK2 #9125, phospho-MEK1/2 (Ser 217/221) #9154, (B) both ERK1 and ERK2: p44/42 MAPK (Erk1/2) #9102 and phospho-ERK1 and ERK2: phospho-p44/42 MAPK (Erk1/2) (Thr202/Tyr204) (D13.14.4E) XP #4370 and (C) Src #2123 and phospho-Src (Tyr 416) #6943. Histone H3 was used as a loading control. *P < 0.05.

Fig. S3. Densitometric analysis of the expression of MEK1, MEK2 and phospho-MEK1/2, in K562-STI-R cells (untreated) and K562-STI-R cells cultured in the presence of 25 lM IM, 25 lM U0126, 25 lM PS-1145,

100 nM dasatinib and combinations of 100 nM dasatinib and 25 lM PS-1145, 25 lM U0126 and 25 lM PS-

1145, 100 nM dasatinib and 25 lM U0126 and 100 nM dasatinib, 25 lM U0126 and 25 lM PS-1145.

Fig. S4. Densitometric analysis of the expression of ERK1/2 and phospho-ERK1/2, in K562-STI-R cells

(untreated) and K562-STI-R cells cultured in the presence of 25 lM IM, 25 lM U0126, 25 lM PS-1145,

100 nM dasatinib, the combination of 100 nM dasatinib and 25 lM PS-1145, the combination of 25 lM U0126 and 25 lM PS-1145, the combination of 100 nM dasatinib and 25 lM U0126, or the combination of 100 nM dasatinib, 25 lM U0126 and 25 lM PS-1145.

Fig. S5. Densitometric analysis of the expression of Src and phospho-Src in K562-STI-R cells (untreated)

and K562-STI-R cells cultured in the presence of 25 lM IM, 25 lM U0126, 25 lM PS-1145, 100 nM

dasatinib, the combination of 100 nM dasatinib and 25 lM PS-1145, the combination of 25 lM U0126 and 25 lM PS-1145, the combination of 100 nM dasatinib and 25 lM U0126, or the combination of 100 nM dasatinib, 25 lM U0126 and 25 lM PS-1145.

Fig. S6. Densitometric analysis of the expression of NF-jB and phospho-NF-jB in (A) K562 and K562-

STI-R cells and (B) in K562-STI-R cells (untreated) and K562-STI-R cells cultured in the presence of

25 lM IM, 25 lM U0126, 25 lM PS-1145, 100 nM dasatinib and combinations of 100 nM dasatinib and

25 lM PS-1145, 25 lM U0126 and 25 lM PS-1145, 100 nM dasatinib and 25 lM U0126 and 100 nM dasatinib, 25 lM U0126 and 25 lM PS-1145.
